# Effects of the antidepressant fluoxetine on pigment dispersion in chromatophores of the common sand shrimp, *Crangon crangon*: repeated experiments paint an inconclusive picture

**DOI:** 10.1007/s10646-020-02272-7

**Published:** 2020-08-28

**Authors:** Alex T. Ford, Eleanor Feuerhelm

**Affiliations:** Institute of Marine Sciences, School of Biological Sciences, University of Portsmouth, Ferry Road, Portsmouth, PO4 9LY UK

**Keywords:** SSRIs Pharmacetuicals, Invertebrates, Ecotoxicology, Pollution, Neuroendocrine

## Abstract

The effects of antidepressants in the environment are starting to generate considerable interest due to the fact that neurotransmitters influence a range of biological processes. Crypsis is an important behavioural and physiological response in many crustaceans modulated by monoamine and pigment dispersing/concentrating hormones. This study aimed to develop a test methodology and investigate the effects of the selective serotonin reuptake inhibitor (SSRI), fluoxetine, on a chromatophore index and overall carapace ‘darkness’ in the common sand shrimp *Crangon crangon*. Adult shrimp were exposed for either 1 h, 1 day or 1 week across a range of nominal fluoxetine concentrations (10 ng/L, 100 ng/L and 1000 ng/L) and the chromatophore index or carapace percentage ‘darkness’ was recorded following 30 min on white and black substrates. These experiments were repeated three times using different specimens. Animals became significantly darker (~20%) on darker background and lighter on light backgrounds as one might expect. However, time periods over which the animals were recorded had a significant impact on the colouration suggesting habituation to laboratory conditions. Fluoxetine exposure came up as a significant factor in two of the three trials for the chromatophore index but the results was inconsistent between trials. There was a high degree of correlation between the chromatophore index and the percentage darkness analyses however, there was no significant effects for fluoxetine exposure with the percentage darkness data. We conclude that the effects on antidepressants on colour change remain inconclusive from these experiments and we discuss potential areas to improve the repeatability of the experiments.

## Introduction

The effects of some pharmaceuticals on the environment have received considerable attention over past years due to effects on wildlife observed at the individual and population level (Halling-Sørensen et al. 1998; Fent et al. 2006; Brausch et al. 2012; Arnold et al. 2014; Saaristo et al. 2018). The concern over the effects of antidepressants in the environment has grown steadily over the past decade due to the fact that neurotransmitters, with which the drugs are designed to interact, control a large variety of biological processes (Guler and Ford 2010; Fong and Ford 2014). Furthermore, recent studies have highlighted effects at very low and environmentally relevant concentrations (Di Poi et al. 2013; De Lange et al. 2006; Bossus et al. 2014; Fong and Hoy 2012; Fong and Molnar 2013). Standard environmental toxicology testing focuses upon mortality, growth and reproductive based endpoints and whilst these have some links with behaviour, these tests would not pick up non-standard endpoints such as disrupted colour change. The ability of an organism to change colour and adapt to its background is critical to its survival, therefore, it is important to develop test methods that can determine if pollutants can impact these novel endpoints.

Many crustaceans have the capacity to change colour over time as a means of remaining cryptic from predators through morphological changes to their carapace and shorter-term physiological changes in the pigment dispersion/concentration of the chromatophores (Keeble and Gamble 1904). In some species the hormonally controlled process of colour change is relatively fast (less than 1 h) whereas others change colour diurnally or seasonally (Detto et al. 2008, Siegenthaler et al. 2017; Green et al. 2019). For example, some planktonic organisms are considered to balance the benefits of transparency with protection of UV damage in shallow depths (Bashevkin et al. 2019). Experiments with the crab, *Paraxanthus barbiger* have shown fish predation rates twice as high when crabs were on plain backgrounds (~60%) as opposed to heterogenous ones (~30%) which were more likely to match their carapace colour or pattern (Manríquez et al. 2008). Furthermore, this study also recorded behavioural responses whereby crabs were more likely to choose a heterogenous background in response to fish predatory cues. This highlights that both the physiological and behavioural responses are important to avoiding predation. Chameleon prawns (*Hippolyte varians*) come in two distinct colour forms (green and red) and display distinct behavioural preference for red and green algae as well as a capacity to change colour between day and night and seasonally to match their algal cover (Green et al. 2019). Failure by an organism to behaviourally or physiologically adapt to its environment as a result of pollution would conceivably result in an increased predation, increased metabolic costs and potential risks through UV radiation.

Antidepressant drugs such as fluoxetine have been detected in the surface water and in wastewater effluent respectively at levels up to 0.54 μg/L and 0.929 μg/L (Brooks et al. 2003; Metcalfe et al. 2010; Styrishave et al. 2011; Silva et al. 2015). Fluoxetine has also been detected in groundwater at 0.056 μg/L (Silva et al. 2015). An increasing number of studies are recording fluoxetine in marine coastal areas therefore the impacts of these drugs need to be determined in non-target organisms. For example, the following studies have observed fluoxetine ranging from 0.58 to 90 ng/L in coastal waters (Pait et al. 2006; Nödler et al. 2014; Birch et al. 2015; Biel-Maeso et al. 2018). Fluoxetine and its metabolite norfluoxetine have been shown to bioaccumulate in several fish species (Arnnok et al. 2017). Uptake of antidepressants by invertebrates and subsequent predation by freshwater fish and duck-billed platypus has suggested these predators maybe getting anywhere between a 30 and 60% human equivalent dose per day through their diet (Richmond et al. 2018).

Neurohormones and neurotransmitters control a wide variety of biological functions within the crustaceans including: reproduction, growth, maturation, larval development, immune function; metabolism, behaviour and colour physiology (Fingerman 1987, 1997; Sarojini et al. 1995; Huber et al. 1997; Cheng et al. 2006). Fong and Ford (2014) recently concluded that because antidepressants interfere with neurohormones and neurotransmitters, they have the potential to effect multiple biological processes including reproduction, growth, metabolism, immunity, feeding, locomotion, colour physiology and behaviour. In their review they posed a number of questions relating to paucity of knowledge in the field of which one included the question do antidepressants impact an organism’s ability to change colour and remain cryptic in their environment?

Di Poi et al. (2014) reported that cuttlefish (*Sepia officinalis*) displayed altered camouflage and sand digging following low exposures to fluoxetine (1–100 ng/L). In this experiment dopamine but not serotonin concentrations in the tissues were significantly different from the control. Bidel et al. (2016a) contrastingly did not observe any impacts on camouflage ability with cuttlefish exposed to fluoxetine but also observed changes to the dopanergic but not serotonergic pathways. Their study also reported decreased cell proliferation in some parts of the cuttlefish brain associated with cognitive and visual processing. Bidel et al. (2016b) using the antidepressant Venlafaxine which targets both serotonin and norepinephrine pathways also found a decrease in camouflage ability following 20 days exposure at 100 ng/L which corresponded with decreases in norepinephrine and increases in some brain regions (vertical and optic lobes).

The sand shrimp (*Crangon crangon*) is an ecologically and commercially important crustacean across Western Europe and can be found from Portugal to Norway (Campos and Van Der Veer 2008). They are known for their capacity to change colour and have been studied as a model organism for neurological control for colour for over a century (Fingerman et al. 1998). Crustacean chromatophores may contain one (monochromatic), two (dichromatic) or several (polychromatic) pigments (Highman and Hill 1977a, b) and were fairly comprehensively studied in the 1970s. In *C. crangon* there are typically different coloured chromatophores within polychromatic chromatosomes which are a mixture of: monochromatic black; dichromatic black-red; trichromatic brown-yellow-red; and tetrachromatic black-white-yellow-red (Highman and Hill 1977a, b). The colour change in *Crangon* species has been commonly used in laboratory teaching experiments (O’Halloran 1990) and recently scientists have been trying to further optimise methods to quantify colour change in *C. crangon* as a tool for answering essential physiological, behavioural and evolutionary questions (Siegenthaler et al. 2017).

Colour regulation within crustaceans is complex and controlled through a number of neurotransmitters which act upon chromatophores sometimes antagonistically within the epithelial tissues (Fingerman 1997). These neurotransmitters control release of pigment concentrating or dispersing hormones in the eye stalks which subsequently act upon the chromatophores. Previously, these have often been named after the colour of the pigments (e.g. red-pigment dispersing hormone; RPDH or red-pigment concentrating hormone; Fingerman and Fingerman 1977) although the evidence for multiple pigment dispersing (PDH) and concentrating (PCH) hormones is yet to be confirmed. Both serotonin and dopamine have been shown to influence pigment dispersing hormone (PDH) whilst dopamine is also known to impact pigment concentrating hormone (PCH). Injection of serotonin into crustaceans evokes the release of PDH creating an increase in the chromatophore pigments throughout the cells in a dose dependant manner (Hanumante and Fingerman 1981, 1983; Fingerman 1985). Norepinephrine has been suggested to control the release of black-pigment dispersing hormone but not RPDH (see Fingerman 1997 and papers therein). Fingerman (1997) highlighted the potential for pollutants to impact colour change in crustaceans although none so far have looked at pharmaceutical pollutants known to disrupt neuroendocrine system. Indeed, Fluoxetine has been used previously to test the mechanistic role of neurotransmitters such as serotonin. Injection by fluoxetine into the fiddler crab (*Uca pugilator*) results in a release of PDH and a darkening on the chromatophores (Fingerman et al. 1981; Hanumante and Fingerman 1981, 1983).

The aim of these experiments was to determine whether the antidepressant fluoxetine could alter the ability of *Crangon crangon* to change their colour when presented with a white background and then transferred to a black background. Based on the previous experiments using Fluoxetine injected in vivo, our working hypothesis was that fluoxetine exposure from water would result in a darkening of the carapace and thus would be darker on white backgrounds and present the least change when transferred to black backgrounds.

## Methods

Specimens of *Crangon crangon* were collected from Hayling Island Beach (Hampshire, UK; 50.790124, −1.023318) using a push net during summer 2011, 2013 and 2014 and kept at the Institute of Marine Science aquarium facilities for several months fed mussel tissue *ad libitum*. Experiments conducted in the winter used specimens collected the previous summer. Aquarium facilities receive natural seawater (pH 8.1) from Langstone Harbour, which is filtered through a 4-weir sedimentation system followed by glass bead and sand filtration at ambient temperatures. The seawater system is connected to heater-chillers and tanks kept were temperature-controlled rooms. The fluoxetine concentrations in Langstone Harbour are not known therefore field collected specimens may have been exposed to effluent periodically from storm water overflows. Between 30 and 60 adult specimens of a similar size (~40–50 mm) were sorted for each of the experiments (Trials 1–3; Table 1).

**Table 1:** Experimental protocol of Fluoxetine exposures across 3 trials to the common sand shrimp, *Crangon crangon*

Experiment	Date	Replicates per treatment	Treatment	Coefficient scores	Image analysis
Trial 1	Dec 2012	10	Control, 10 ng/L and 1000 ng/L	Yes	No
Trial 2	Feb 2014	15	Control, 10 ng/L, 100 ng/L and 1000 ng/L	Yes	Yes
Trial 3	Oct 2014	15	Control, 10 ng/L, 100 ng/L and 1000 ng/L	Yes	Yes

Fluoxetine hydrochloride (CAS Number 56296-78-7) was purchased from Sigma-Aldrich and made into 2 mg/L stock solutions before being serially diluted to nominal concentrations of 10 ng/L, 100 ng/L or 1000 ng/L in natural filtered seawater. Stock was kept in foil and refrigerated during the course of the 1 week exposures and water changes on day 3 or 4 of the experiments. Individual shrimp were kept in 200 ml crystallising dishes and exposed to 10 ng/L, 100 ng/L or 1000 ng/L fluoxetine for 1 week and compared to control animals (*n* = 10–15 per treatment). Animals were kept at 10 ± 1 °C in a 12:12 light:dark cycle.

**Fig. 1:**
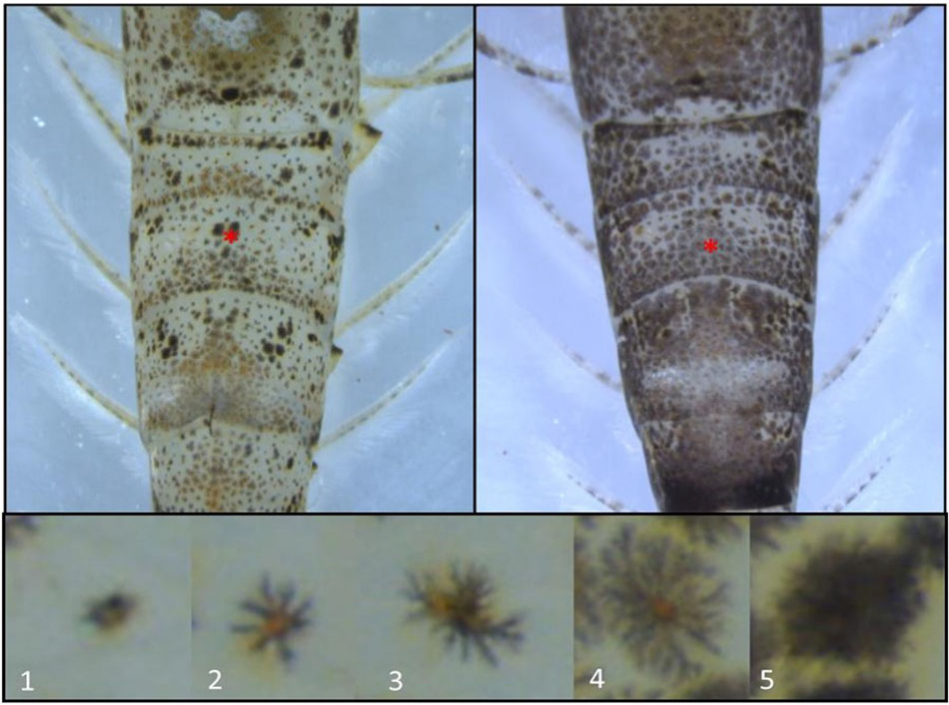
Example *Crangon crangon* with light (left) and dark (right) patterning. Bottom plate indicates the scoring of the chromatophores 1–5. Red Star (position of the 2nd pleura)

After 1 h, 1 day and 1 week exposures the crystallising dishes were covered with white paper covering the sides and bottom of the containers. After 30 min and a digital photo taken of the second abdominal segment (pleura; Fig. 1). The white paper was removed and replaced immediately with black paper and 2nd photo was recorded following another 30 min. The digital photographs were randomly numbered so that the recorder was not aware of the control and treatment groups and the chromatophore index was recorded for every single chromatophore within the 2nd abdominal segment. The chromatophores were graded from (1) small and very concentrated to (5) large and very dilated. These experiments were repeated across 3 separate trials and data recorded by the same person.

Following this grading of chromatophore dispersion, a chromatophore coefficient was calculated in methods adapted from O’Halloran (1990). The methods by O’Halloran (1990) were based on choosing 20 chromatophores with a minimum and maximum score of between 20 and 100, whereas we choose to look at the total chromatophores within the 2nd abdominal segment. To account for variation in chromatophores per segment and size differences in specimens the eventual score was standardised to the total number of chromatophores (Tc) and expressed back a proportion of 20.

$${\mathrm{Chromatophore}}\;{\mathrm{Coefficient}} \,=\, \left( {\left[ {n1/Tc} \right] \,\times\, 20} \right) \,+\, \left( {\left[ {n2/Tc} \right] \,\times\, 20} \right) \,+\, \left( {\left[ {n3/Tc} \right] \,\times\, 20} \right) \,+\, \left( {\left[ {n4/Tc} \right] \,\times\, 20} \right) \,+\, \left( {\left[ {n5/Tc} \right] \,\times\, 20} \right).$$

where *n*1 to 5 = the number of chromatophore cells in stage 1 to 5 for an individual. This value is divided by the total number of chromatophores and multiplied by 20. Following the calculation of *n*1 to *n*5 these values are added to generate the chromatophore score which should fall between 20 (very light coloured) and 100 (very dark coloured).

ImageJ (https://imagej.nih.gov/ij/) was used to calculate the percentage area of the 2nd abdominal segment covered by chromatophore cells for trials 2 and 3 (Table 1). This was done by drawing around the second abdomen segment, before converting it to 8-bit to make the image greyscale (Supplementary Fig. 1). A median filter was then added to smoothen the image with a 2 pixel radius. The threshold value was then adjusted until pixels covered the chromatophore cells. The particles were then analysed to indicate the percentage coverage.

Data was analysed by of means of a repeated measure 3-way Analysis of Variance (ANOVA) with a Greenhouse-Geisser adjustment whereby both chromatophore index scores and percentage cover data were natural log transformed to confirm with normality assumptions. The repeated measure was either the chromatophore index or percent cover on 1st the white and then the black background. The fixed factors were concentration (Control, 10 ng/L, 100 ng/L or 1000 ng/L Fluoxetine), trial (1, 2 or 3) and Exposure Time (1 h, 1 day and 1 week). Additional post hoc pairwise (Tukey) comparisons were conducted adjusting using a Bonferroni correction. All interactions were observed using interaction plots and sequential removal of fixed factors. The relationship between chromatophore scores and percentage cover were analysed by Pearson’s correlation.

## Results

### Chromatophore index

**Fig. 2:**
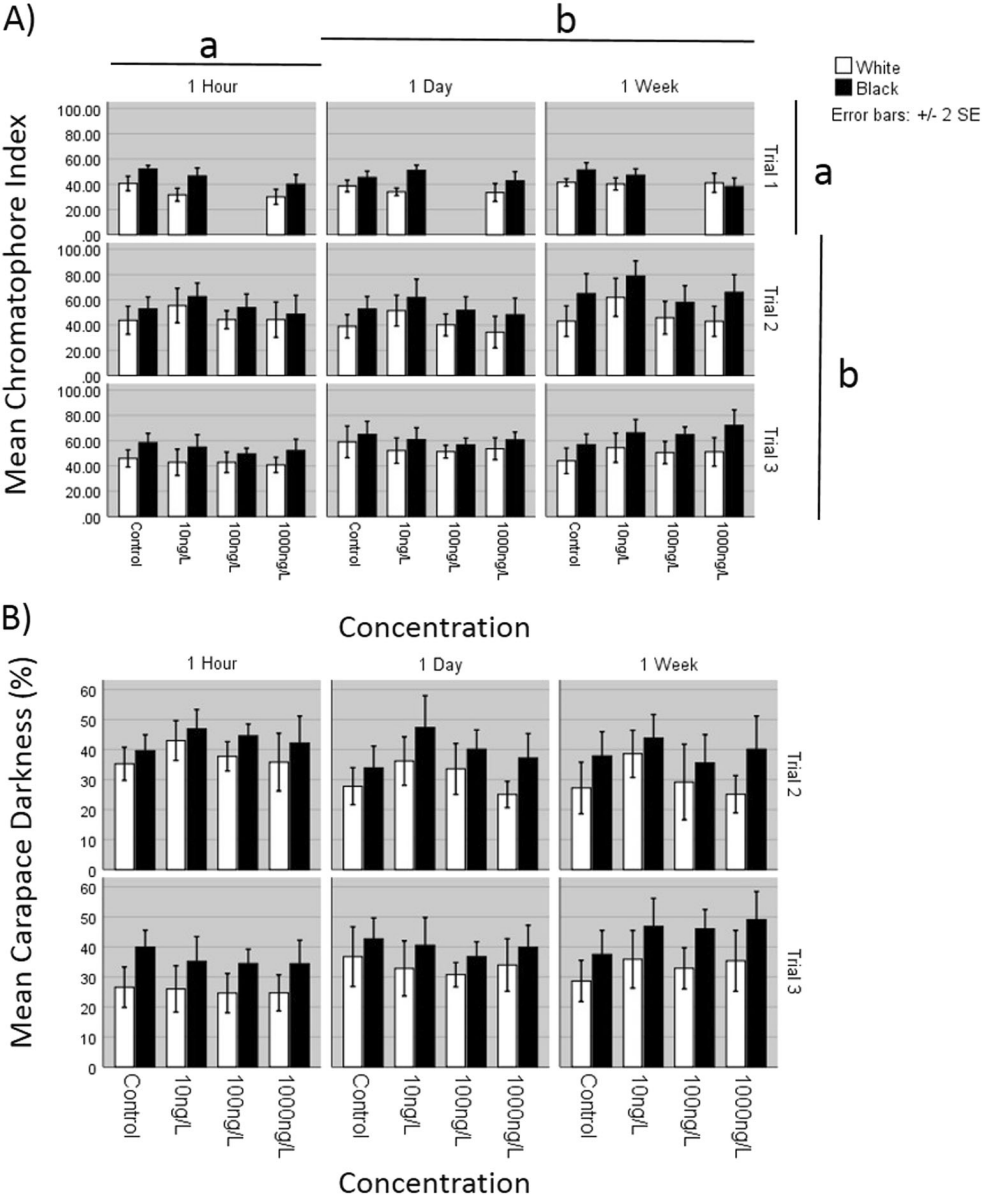
Trails 1–3 (**a**, **b**) of mean chromatophore index scores and carapace darkness percentage of *Crangon crangon* kept on white and back backgrounds following exposure to Fluoxetine (Control, 10, 100 and 1000 ng/L). Different letters denote significant differences between the times and the trails (*p* < 0.05)

A repeated measures ANOVA with a Greenhouse-Geisser correction determined that the mean chromatophore index was significantly greater on black compared to white backgrounds (*F* = 188.823, df = 1, *p* < 0.001, *η*_p_^2^ = 0.376; Supplementary Table 2; Fig. 2). There was, however, a significant interaction between background with the different trials and exposure times (*F* = 5.105, df = 4, *p* = 0.001, *η*_p_^2^ = 0.061; Greenhouse-Geisser adjusted). Sequential removal of different trials indicated there were smaller changes between the white to black backgrounds during trial 1 compared to the other two trials.

Between subject analyses revealed there was a significant effect of fluoxetine concentration (*F* = 5.131, df = 3, *p* = 0.002). There was a significant interaction between the trials and concentration (*F* = 3.912, df = 5, *p* = 0.002) Sequential removal of the trials revealed no significant differences between concentrations in trial 3 (*p* > 0.05). In trial 1, the chromatophore index was significantly lower for 1000 ng/L (*p* < 0.05) compared to the controls but not the 100 ng/L. Conversely, in trial 2 the 10 ng/L had significantly higher chromatophore indices from the controls (*p* < 0.05) but no other concentrations. There were significant differences between the different trials (*F* = 27.614, df = 2, *p* < 0.001). Post hoc analysis highlighted a significantly lower chromatophore scores recorded in the 1st trial compared to both trials 2 and 3 (*p* < 0.05; Supplementary Table 2). Exposure times also had a significant impact on chromatophore concentrations (*F* = 5.628, df = 2, *p* = 0.004) with overall scores increasing with exposure period and 1 week scores significantly higher than both the 1 h and 1 day scores (*p* > 0.05; Supplementary Table 2).

Interaction also occurred between the trial and exposure time (*F* = 3.074, df = 4, *p* = 0.017; see Supplementary Table 2) which may have occurred due to some unusual scores recorded after 1 h during trial 3. No interaction was observed between concentration and exposure time (*p* > 0.05) and any 3 way interaction between trial, concentration and exposure time (*p* > 0.05).

### Percentage cover

The analysis of percentage cover was only analysed for trials 2 and 3. Similarly with the chromatophore index, there was a significant increase in the darkening of the carapace in shrimp on the black as opposed to the white backgrounds (*F* = 177.574, df = 1, *p* < 0.001, *η*_p_^2^ = 0.431; Supplementary Table 3; Fig. 2). There was also an interaction between the background colour and the number of the trial and exposure time (*F* = 4.595, df = 2, *p* = 0.011, *η*_p_^2^ = 0.038).

Between subject analyses revealed there was no significant effect of fluoxetine concentrations on the overall percentage dark cover (*F* = 1.997, df = 3, *p* = 0.115) or any difference between the two trials (*F* = 1.828, df = 1, *p* = 0.178; Supplementary Table 3). The was also no significant difference in the percentage dark areas over the exposure time (*F* = 0.013, df = 2, *p* = 0.987). However, there was a significant interaction between trial number and time (*F* = 11.255, df = 2, *p* < 0.001) with coverage scores getting lower over time in trial 2 but greater over time in trial 3. No interaction was observed between concentration and exposure time (*p* > 0.05) or between the different trials and concentrations (*p* > 0.05). Neither was there a 3 way interaction between trial, concentration and exposure time (*p* > 0.05).

**Fig. 3:**
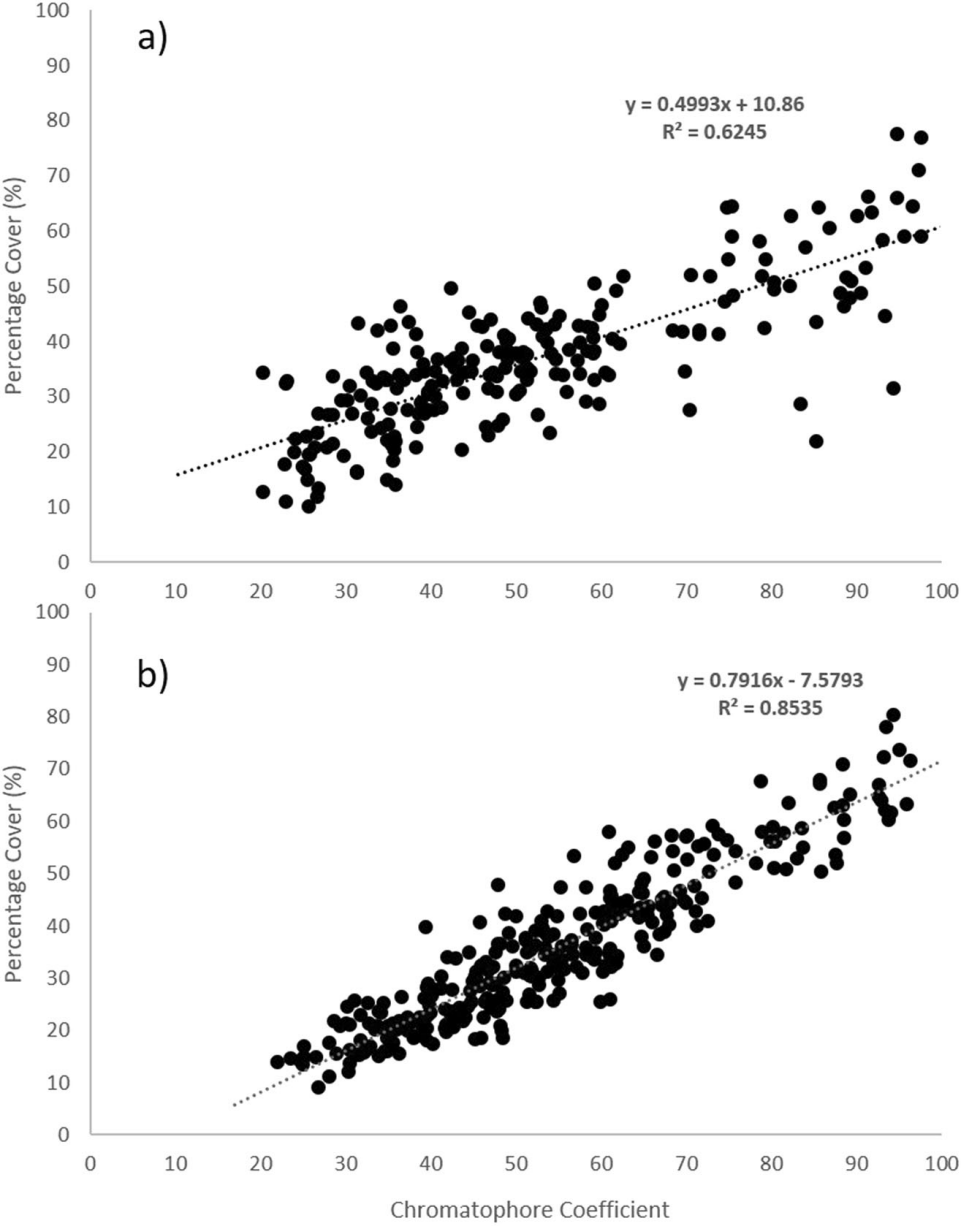
Relationship between chromatophore coefficient scores and percentage dark cover on *Crangon crangon* exposure to Fluoxetine (Control, 10, 100 and 1000 ng/L). **a** Trial 2 and **b** Trial 3

The chromatophore indices generated by ‘blind’ assessment of chromatophore dispersion were compared with those generated for percentage dark cover using image analysis software converting photos in binary black and white images. For both trials 2 and 3 these two measurements significantly and highly correlated (Pearson’s Trial 2: 0.793; *p* < 0.001; *R*^2^ = 0.6245: Trial 3 0.923; *p* < 0.001; *R*^2^ = 0.8535 Fig. 3).

## Discussion

Given the role that some monoamines have on pigment dispersing and concentrating hormones in crustaceans (Fingerman 1966, 1997), we hypothesised those serotonin-based modulators such as the antidepressants might interfere with a crustacean’s ability to change colour. The ability to change colour is very important in terms of survival from predation and signalling to fellow conspecifics aggression or mate status (Stevens and Merilaita 2008).

The results of this study found that in two of the three trials for the chromatophore index, the treatment groups had significantly different chromatophore dispersion from the controls. However, and importantly, the lower chromatophore scores and percentage cover recorded in trial 1 1000 ng/L were in direct contrast to the higher measurements recorded for 10 ng/L in trial 2. Given we found no significant effects of treatment in our 3rd trial and no significant effects using the percentage darkness parameter we feel these results should be treated with caution at this stage. However, they do help illustrate some important issues around repeatability within ecotoxicology especially when developing novel assays.

The chromatophore measurements graded by human eye are arguably more subjective but were remarkably similar to the overall darkness measured by image analysis and those by Siegenthaler et al. (2018). Coupled with the fact that all these experiments were conducted ‘blind’, these results give us some confidence that the different measurements recorded from the experiments were ‘real’ physiological colour differences in the observed animals and not variability with the measurements themselves. Therefore, one may speculate that the variable responses observed between the 3 trials maybe due to variability in the biological status of the organisms used.

Recently, Siegenthaler et al. (2018) reported considerable intra- and inter-individual variation in the colour patterning on *C. crangon* that they mention demonstrates a complex balance of behavioural plasticity and environmental adaption. These authors mentioned the time of day and illumination are confounding factors in the colour change adaption of *C. crangon*. In our study, specimens were kept in incubators under controlled lighting and temperature and whilst the experiment recording may have taken place at different times between trials, we believe any tidal rhythms would have abated through long laboratory acclimation prior to experiments. Our experiments were undertaken at different times of the year so one possible cause of variation could be the seasonal variation in response to serotonin modulators such as antidepressants. An additional source of variation could also have been the variable daylight whilst photos were recorded under the microscope. Unfortunately, lux recordings within the laboratory were not undertaken during these experiments and should be considered for future experiments.

**Fig. 4:**
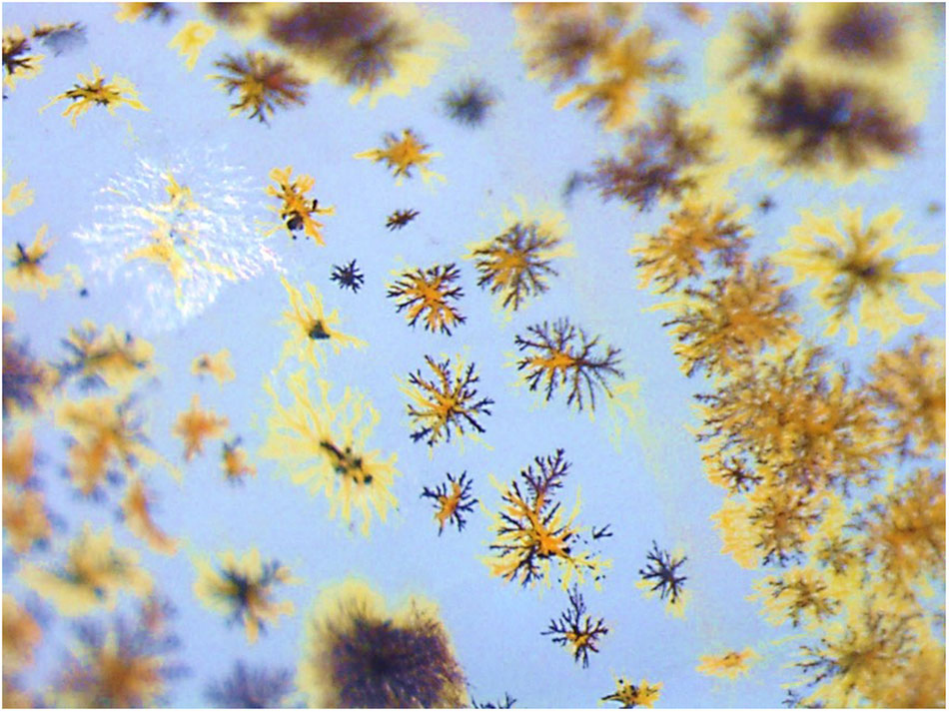
Typical variation in chromatophore colours along the carapace of *Crangon crangon*

The different neurotransmitters in crustaceans have been suggested to modulate different colour pigments, sometimes and in antagonistic ways (Fingerman 1985). Our working hypothesis was that fluoxetine, because it acts on PDH, would result in the chromatophore indices become greater and the increase the overall darkness. However, the degree to which fluoxetine impacts on PDH will no doubt depend on the exposure dose and period. Fluoxetine is known as a promiscuous compound, binding to several neurotransmitter receptors (Stahl 1998) as well as the serotonin reuptake transporter proteins (SERT). Therefore, it is quite possible that depending on the concentrations used, there may be antagonistic impacts of multiple receptor binding, resulting in the dispersal of some pigments and concentrating of others. The recording method used in this paper only noted the overall chromatophore expression and did not single out the red chromatophores for which may have been more responsive to the exposure compounds (Fig. 4). Therefore, further method development, especially if using image analysis should include a means of filtering out the relevant wavelengths.

Serotonin modulates many biological responses in crustaceans including reproduction, moulting, metabolism as well as colour change and behaviour. Whilst just speculation, it is possible that the *C. crangon* react differently to fluoxetine at different times of the year when their own physiological status in terms of reproduction, metabolism and moulting can vary. Some specimens were conspicuously very dark or very light on the opposing backgrounds which may be intraspecific variability but also could have indicated profound changes in their internal biology such as may occur during moulting and/or sex changes. Detto et al. (2008) reported that the most dramatic changes in fiddler crab (*Uca capricornis*) colour patterns were caused by moulting when measuring social and environmental impacts in colour. These shrimps are sequential hermaphrodites and whilst we did not record the sex in these individuals the sizes used indicated we most likely were working with females. Stock solutions for each experiment were made up in exactly the same way but clearly when using nominal concentrations its unknown whether the actual water concentrations varied between experiments. Previous experiments in our labs have demonstrated equitability between nominal and actual concentrations and breakdown rates in line with other studies (De Castro-Català et al. 2017).

We know from our repeated experiments that the animals respond similarly to the experimental setup i.e. their responses to dark arenas resulted in an overall 20% increase in chromatophore scores or percentage chromatophore cover. This relatively quick capacity to change colour enables them to stay camouflaged and adapt to different coloured sandy substrates. Siegenthaler et al. (2018) similarly found specimens became approximately 20% darker on black vs. white backgrounds. We know that the time the animals were kept in their individual chambers (1 h to 1 week) resulted in different chromatophore and percentage cover scores suggested either some habituation to test conditions or reduced ‘health’ of the animals under laboratory conditions. Siegenthaler et al. (2018) found that specimens kept over a 21 day period on a black background reduced their capacity to change colour to white backgrounds. This was in contrast to specimens that were interchanged between white and black background through the 21 day period.

Whilst it is difficult to conclude too much at this stage as to our overall effects of antidepressants on crustacean colour change, this study has highlighted how three repeated experiments can yield quite variable biological responses. We know that the ability of some crustaceans to adapt its colour to match its background is critical for their survival (Manríquez et al. 2008). Therefore, there is a clear need to better understand the baseline biological responses of these organisms to changing environments so that the effects of pollution on colour change can be answered.

## Supplementary information

Supplementary Information
