# Supplementary material for: Effects of the antidepressant fluoxetine on pigment dispersion in chromatophores of the common sand shrimp, Crangon crangon: repeated experiments paint an inconclusive picture
Source: Ecotoxicology. 2020 Aug 28;29(9):1368–76. doi: 10.1007/s10646-020-02272-7 (PMC7581581; doi:10.1007/s10646-020-02272-7)
Supplement: Supplementary file 1 — Supplementary Information [file 10646_2020_2272_MOESM1_ESM.docx]

Supplementary

Supplementary Table 1: Calculation of chromatophore coefficient. The number of chromatophores of a given stage is given as a proportion of the total and standardised to 20. The standardised score is multiple by the chromatophore stage and subsequently summed for each chromatophore stage.

| Shrimp | Chromatophore Stage | No. of Chromatophores | Chromatophore Score = (No.chromatophores/Total chromatophores)*20 | Chromatophore coefficient = Chromatophore Score*chromatophore stage |
| --- | --- | --- | --- | --- |
| #1 | 1 | 71 | (71/104)*20 = 13.7 | 1 x 13.7 |
|  | 2 | 26 | (26/104)*20 = 5 | 2 x 5 |
|  | 3 | 6 | (6/104)*20 = 1.2 | 3 x 1.2 |
|  | 4 | 1 | (1/104)*20 = 0.2 | 4 x 0.2 |
|  | 5 | 0 | (0/104)*20 = 0 | 5 x 0 |
| Total |  | 104 |  | 27.9 |

Supplementary Table 2: Statistical output from 3-way repeated measure ANOVA for chromatophore index in *Crangon crangon* following 30 minutes transfer from white to back background and across different time periods (1 hour, 1 day and 1 week) and Fluoxetine exposures (0-1000ng/L).

| Source | | Type III Sum of Squares | df | Mean Square | F | Sig. | Partial Eta Squared | Observed Power^a^ |
| --- | --- | --- | --- | --- | --- | --- | --- | --- |
| Background | Sphericity Assumed | 8.944 | 1 | 8.944 | 188.823 | .000 | .376 | 1.000 |
|  | Greenhouse-Geisser | 8.944 | 1.000 | 8.944 | 188.823 | .000 | .376 | 1.000 |
| Background * Trial | Sphericity Assumed | .083 | 2 | .042 | .878 | .417 | .006 | .201 |
|  | Greenhouse-Geisser | .083 | 2.000 | .042 | .878 | .417 | .006 | .201 |
| Background * Concentration | Sphericity Assumed | .045 | 3 | .015 | .314 | .815 | .003 | .111 |
|  | Greenhouse-Geisser | .045 | 3.000 | .015 | .314 | .815 | .003 | .111 |
| Background * Time | Sphericity Assumed | .014 | 2 | .007 | .144 | .866 | .001 | .072 |
|  | Greenhouse-Geisser | .014 | 2.000 | .007 | .144 | .866 | .001 | .072 |
| Background * Trial * Concentration | Sphericity Assumed | .335 | 5 | .067 | 1.413 | .219 | .022 | .497 |
|  | Greenhouse-Geisser | .335 | 5.000 | .067 | 1.413 | .219 | .022 | .497 |
| Background * Trial * Time | Sphericity Assumed | .967 | 4 | .242 | 5.105 | .001 | .061 | .965 |
|  | Greenhouse-Geisser | .967 | 4.000 | .242 | 5.105 | .001 | .061 | .965 |
| Background * Concentration * Time | Sphericity Assumed | .122 | 6 | .020 | .429 | .859 | .008 | .177 |
|  | Greenhouse-Geisser | .122 | 6.000 | .020 | .429 | .859 | .008 | .177 |
| Background * Trial * Concentration * Time | Sphericity Assumed | .296 | 10 | .030 | .624 | .793 | .019 | .327 |
|  | Greenhouse-Geisser | .296 | 10.000 | .030 | .624 | .793 | .019 | .327 |

Between Subject Effects

| Source | Type III Sum of Squares | df | Mean Square | F | Sig. | Partial Eta Squared | Observed Power^a^ |
| --- | --- | --- | --- | --- | --- | --- | --- |
| Intercept | 9402.729 | 1 | 9402.729 | 70266.337 | .000 | .996 | 1.000 |
| Trial | 7.390 | 2 | 3.695 | 27.614 | **.000** | .150 | 1.000 |
| Concentration | 2.060 | 3 | .687 | 5.131 | **.002** | .047 | .921 |
| Time | 1.506 | 2 | .753 | 5.628 | **.004** | .035 | .858 |
| Trial * Concentration | 2.618 | 5 | .524 | 3.912 | **.002** | .059 | .944 |
| Trial * Time | 1.646 | 4 | .411 | 3.074 | **.017** | .038 | .807 |
| Concentration * Time | .621 | 6 | .103 | .773 | .591 | .015 | .306 |
| Trial * Concentration * Time | .631 | 10 | .063 | .472 | .908 | .015 | .245 |
| Error | 42.018 | 314 | .134 |  |  |  |  |
|  |  |  |  |  |  |  |  |

Pairwise Comparisons (Bonferroni corrected) for chromatophore index and different trials

| (I) Trial | (J) Trial | Mean Difference (I-J) | Std. Error | Sig.^d^ | 95% Confidence Interval for Difference^d^ | |
| --- | --- | --- | --- | --- | --- | --- |
|  |  |  |  |  | Lower Bound | Upper Bound |
| Trial 1 | Trial 2 | -.188^*,b^ | .037 | .000 | -.278 | -.099 |
|  | Trial 3 | -.259^*,b^ | .035 | .000 | -.343 | -.176 |
| Trial 2 | Trial 1 | .188^*,c^ | .037 | .000 | .099 | .278 |
|  | Trial 3 | -.071 | .033 | .095 | -.150 | .008 |
| Trial 3 | Trial 1 | .259^*,c^ | .035 | .000 | .176 | .343 |
|  | Trial 2 | .071 | .033 | .095 | -.008 | .150 |
| Based on estimated marginal means | | | | | | |
| *. The mean difference is significant at the .05 level. | | | | | | |
| b. An estimate of the modified population marginal mean (I). | | | | | | |
| c. An estimate of the modified population marginal mean (J). | | | | | | |
| d. Adjustment for multiple comparisons: Bonferroni. | | | | | | |

Pairwise Comparisons (Bonferroni corrected) for chromatophore index and different concentrations

| (I) Conc | (J) Conc | Mean Difference (I-J) | Std. Error | Sig.^d^ | 95% Confidence Interval for Difference^d^ | |
| --- | --- | --- | --- | --- | --- | --- |
|  |  |  |  |  | Lower Bound | Upper Bound |
| Control | 10ng/L | -.050 | .038 | 1.000 | -.150 | .051 |
|  | 100ng/L | -.034^a^ | .044 | 1.000 | -.152 | .084 |
|  | 1000ng/L | .093 | .038 | .088 | -.008 | .193 |
| 10ng/L | Control | .050 | .038 | 1.000 | -.051 | .150 |
|  | 100ng/L | .016^a^ | .044 | 1.000 | -.101 | .133 |
|  | 1000ng/L | .142^*^ | .037 | .001 | .043 | .242 |
| 100ng/L | Control | .034^c^ | .044 | 1.000 | -.084 | .152 |
|  | 10ng/L | -.016^c^ | .044 | 1.000 | -.133 | .101 |
|  | 1000ng/L | .127^*,c^ | .044 | .025 | .010 | .243 |
| 1000ng/L | Control | -.093 | .038 | .088 | -.193 | .008 |
|  | 10ng/L | -.142^*^ | .037 | .001 | -.242 | -.043 |
|  | 100ng/L | -.127^a,*^ | .044 | .025 | -.243 | -.010 |
| Based on estimated marginal means | | | | | | |
| *. The mean difference is significant at the .05 level. | | | | | | |
| a. An estimate of the modified population marginal mean (J). | | | | | | |
| c. An estimate of the modified population marginal mean (I). | | | | | | |
| d. Adjustment for multiple comparisons: Bonferroni. | | | | | | |

Pairwise Comparisons (Bonferroni corrected) for chromatophore index and different times

| (I) Time | (J) Time | Mean Difference (I-J) | Std. Error | Sig.^d^ | 95% Confidence Interval for Difference^d^ | |
| --- | --- | --- | --- | --- | --- | --- |
|  |  |  |  |  | Lower Bound | Upper Bound |
| 1 Hour | 1 Day | -.044^a,b^ | .034 | .612 | -.126 | .039 |
|  | 1 Week | -.120^a,b,*^ | .035 | .002 | -.204 | -.037 |
| 1 Day | 1 Hour | .044^a,b^ | .034 | .612 | -.039 | .126 |
|  | 1 Week | -.077^a,b^ | .035 | .085 | -.161 | .007 |
| 1 Week | 1 Hour | .120^a,b,*^ | .035 | .002 | .037 | .204 |
|  | 1 Day | .077^a,b^ | .035 | .085 | -.007 | .161 |
| Based on estimated marginal means | | | | | | |
| *. The mean difference is significant at the .05 level. | | | | | | |
| a. An estimate of the modified population marginal mean (I). | | | | | | |
| b. An estimate of the modified population marginal mean (J). | | | | | | |
| d. Adjustment for multiple comparisons: Bonferroni. | | | | | | |

Supplementary Table 3: Statistical output from 3 Way repeated measure ANOVA for percentage carapace darkness (2nd Pleura) in *Crangon crangon* following 30 minutes transfer from white to back background and across different time periods (1 hour, 1 day and 1 week) and Fluoxetine exposures (0-1000ng/L).

| Source | | Type III Sum of Squares | df | Mean Square | F | Sig. | Partial Eta Squared | Observed Power^a^ |
| --- | --- | --- | --- | --- | --- | --- | --- | --- |
| Background | Sphericity Assumed | 9.382 | 1 | 9.382 | 177.574 | .000 | .431 | 1.000 |
|  | Greenhouse-Geisser | 9.382 | 1.000 | 9.382 | 177.574 | .000 | .431 | 1.000 |
| Background * Trial | Sphericity Assumed | .134 | 1 | .134 | 2.538 | .113 | .011 | .355 |
|  | Greenhouse-Geisser | .134 | 1.000 | .134 | 2.538 | .113 | .011 | .355 |
| Background * Concentration | Sphericity Assumed | .160 | 3 | .053 | 1.012 | .388 | .013 | .274 |
|  | Greenhouse-Geisser | .160 | 3.000 | .053 | 1.012 | .388 | .013 | .274 |
| Background * Time | Sphericity Assumed | .193 | 2 | .096 | 1.826 | .163 | .015 | .379 |
|  | Greenhouse-Geisser | .193 | 2.000 | .096 | 1.826 | .163 | .015 | .379 |
| Background * Trial * Concentration | Sphericity Assumed | .105 | 3 | .035 | .660 | .578 | .008 | .188 |
|  | Greenhouse-Geisser | .105 | 3.000 | .035 | .660 | .578 | .008 | .188 |
| Background * Trial * Time | Sphericity Assumed | .486 | 2 | .243 | 4.595 | .011 | .038 | .774 |
|  | Greenhouse-Geisser | .486 | 2.000 | .243 | 4.595 | .011 | .038 | .774 |
| Background * Concentration * Time | Sphericity Assumed | .184 | 6 | .031 | .581 | .745 | .015 | .231 |
|  | Greenhouse-Geisser | .184 | 6.000 | .031 | .581 | .745 | .015 | .231 |
| Background * Trial * Concentration * Time | Sphericity Assumed | .137 | 6 | .023 | .431 | .857 | .011 | .177 |
|  | Greenhouse-Geisser | .137 | 6.000 | .023 | .431 | .857 | .011 | .177 |

| Source | Type III Sum of Squares | df | Mean Square | F | Sig. | Partial Eta Squared | Observed Power^a^ |
| --- | --- | --- | --- | --- | --- | --- | --- |
| Intercept | 6092.257 | 1 | 6092.257 | 27740.377 | .000 | .992 | 1.000 |
| Trial | .401 | 1 | .401 | 1.828 | .178 | .008 | .270 |
| Concentration | 1.316 | 3 | .439 | 1.997 | .115 | .025 | .510 |
| Time | .006 | 2 | .003 | .013 | .987 | .000 | .052 |
| Trial * Concentration | 1.409 | 3 | .470 | 2.138 | .096 | .027 | .541 |
| Trial * Time | 4.944 | 2 | 2.472 | 11.255 | **.000** | .088 | .992 |
| Concentration * Time | .617 | 6 | .103 | .468 | .831 | .012 | .190 |
| Trial * Concentration * Time | .819 | 6 | .136 | .621 | .713 | .016 | .246 |
| Error | 51.390 | 234 | .220 |  |  |  |  |

Pairwise Comparisons (Bonferroni corrected) for percentage carapace darkness and different trials

| (I) Trial | (J) Trial | Mean Difference (I-J) | Std. Error | Sig.^a^ | 95% Confidence Interval for Difference^a^ | |
| --- | --- | --- | --- | --- | --- | --- |
|  |  |  |  |  | Lower Bound | Upper Bound |
| Trial 2 | Trial 3 | .057 | .042 | .178 | -.026 | .140 |
| Trial 3 | Trial 2 | -.057 | .042 | .178 | -.140 | .026 |
| Based on estimated marginal means | | | | | | |
| a. Adjustment for multiple comparisons: Bonferroni. | | | | | | |

Pairwise Comparisons (Bonferroni corrected) for percentage carapace darkness and different concentrations

| (I) Conc | (J) Conc | Mean Difference (I-J) | Std. Error | Sig.^a^ | 95% Confidence Interval for Difference^a^ | |
| --- | --- | --- | --- | --- | --- | --- |
|  |  |  |  |  | Lower Bound | Upper Bound |
| Control | 10ng/L | -.123 | .059 | .216 | -.279 | .032 |
|  | 100ng/L | -.037 | .061 | 1.000 | -.201 | .126 |
|  | 1000ng/L | .000 | .058 | 1.000 | -.155 | .155 |
| 10ng/L | Control | .123 | .059 | .216 | -.032 | .279 |
|  | 100ng/L | .086 | .061 | .961 | -.076 | .248 |
|  | 1000ng/L | .123 | .058 | .206 | -.031 | .277 |
| 100ng/L | Control | .037 | .061 | 1.000 | -.126 | .201 |
|  | 10ng/L | -.086 | .061 | .961 | -.248 | .076 |
|  | 1000ng/L | .037 | .061 | 1.000 | -.124 | .199 |
| 1000ng/L | Control | .000 | .058 | 1.000 | -.155 | .155 |
|  | 10ng/L | -.123 | .058 | .206 | -.277 | .031 |
|  | 100ng/L | -.037 | .061 | 1.000 | -.199 | .124 |
| Based on estimated marginal means | | | | | | |
| a. Adjustment for multiple comparisons: Bonferroni. | | | | | | |

Pairwise Comparisons (Bonferroni corrected) for percentage carapace darkness and different times

| (I) Time | (J) Time | Mean Difference (I-J) | Std. Error | Sig.^a^ | 95% Confidence Interval for Difference^a^ | |
| --- | --- | --- | --- | --- | --- | --- |
|  |  |  |  |  | Lower Bound | Upper Bound |
| 1 Hour | 1 Day | -.008 | .051 | 1.000 | -.131 | .115 |
|  | 1 Week | -.005 | .052 | 1.000 | -.130 | .120 |
| 1 Day | 1 Hour | .008 | .051 | 1.000 | -.115 | .131 |
|  | 1 Week | .003 | .052 | 1.000 | -.122 | .129 |
| 1 Week | 1 Hour | .005 | .052 | 1.000 | -.120 | .130 |
|  | 1 Day | -.003 | .052 | 1.000 | -.129 | .122 |
| Based on estimated marginal means | | | | | | |
| a. Adjustment for multiple comparisons: Bonferroni. | | | | | | |


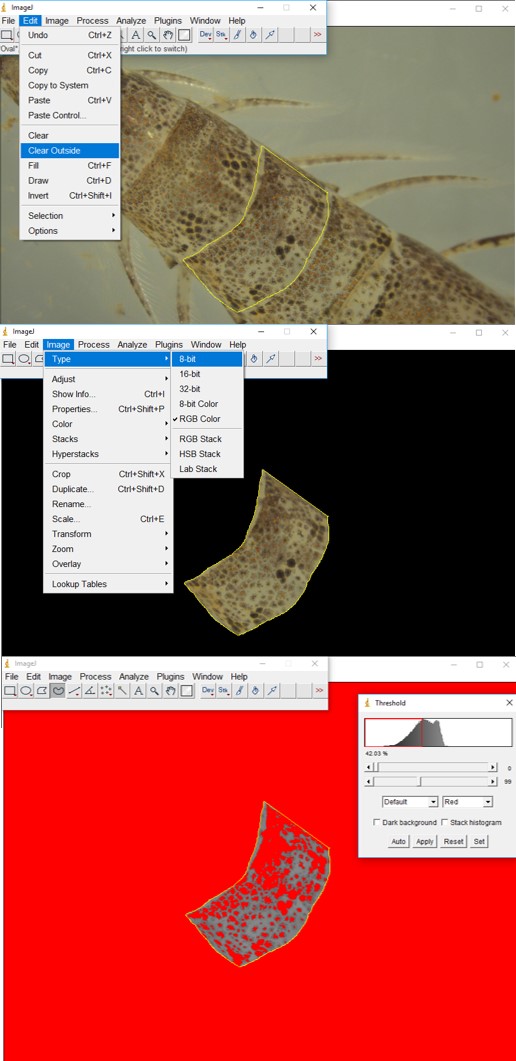


Supplementary Figure 1: Screen shots of ImageJ during the image processing to determine percentage pleura 2 dark coverage in *Crangon crangon* exposed to Fluoxetine.
